# Supplementary material for: Beverage Intake and Associated Nutrient Contribution for Aboriginal and Torres Strait Islander Australians: Secondary Analysis of a National Dietary Survey 2012–2013
Source: Nutrients. 2022 Jan 24;14(3):507. doi: 10.3390/nu14030507 (PMC8839560; doi:10.3390/nu14030507)
Supplement: Supplementary file 1 [file nutrients-14-00507-s001.zip › nutrients-1514291-supplementary.pdf]

Supplementary Table S1: Mean beverage intake (g) and 10th, 90th percentile for those who consumed any of the beverage category on the day of the National Aboriginal and Torres Strait Islander Nutrition and Physical Activity Survey (NATSINPAS) 2012-13 by remoteness category.

	Metro and Regional		Remote		P	Total	
**Age Group (yrs)**	**Mean* (g) (10th-90th %ile)**	**N** (%)**	**Mean* (g) (10th-90th %ile)**	**N** (%)**	**Mean*** (Non-remote vs Remote)**	**Mean* (g) (10th-90th %ile)**	**N** (%)**
**All Beverages**							
**2-3**	1161 (401-1878)	100 (98)	857 (328-1416)	135 (97.8)	0.008	1099 (400-1800)	235 (97.9)
**4-8**	1169 (508-2000)	213 (99.5)	1250 (483-2189)	300 (99.7)	0.505	1183 (500-2000)	513 (99.6)
**9-13**	1398 (629-2388)	175 (99.4)	1750 (588-3350)	229 (98.3)	0.001	1468 (628-2556)	404 (98.8)
**14-18**	2151 (716-4053)	149 (97.4)	2002 (590-3580)	177 (98.9)	0.497	2122 (614-4051)	326 (98.2)
**2-18**	1489 (563-2660)	637 (98.8)	1552 (473-3045)	841 (98.8)	0.198	1501 (535-2697)	1478 (98.8)
**19-30**	2419 (946-4977)	332 (100)	2524 (875-4465)	390 (100)	0.796	2440 (936-4500)	722 (100)
**31-50**	2592 (1052-4722)	481 (99.8)	2666 (864-5540)	616 (100)	0.571	2609 (1020-4829)	1097 (99.9)
**51-70**	2220 (751-3901)	300 (100)	2102 (758-3944)	422 (100)	0.438	2192 (751-3936)	722 (100)
**71+**	1590 (540-2633)	33 (100)	2149 (819-3498)	38 (100)	0.136	1691 (540-2881)	71 (100)
**19+**	2435 (962-4295)	1146 (99.9)	2486 (835-4465)	1466 (100)	0.543	2446 (936-4396)	2612 (100)
**Total**	2042 (716-3820)	1783 (99.5)	2137 (639-4000)	2307 (99.6)	0.285	2061 (700-3870)	4090 (99.5)
**Tea**							
**2-3**	-****	-****	-****	-****	-****	-****	-****
**4-8**	-****	-****	246 (62-683)	53 (17.6)	-****	245 (80-327)	61 (11.8)
**9-13**	-****	-****	414 (84-678)	62 (26.6)	-****	377 (86-678)	70 (17.1)
**14-18**	-****	-****	372 (84-977)	70 (39.1)	-****	411 (150-731)	85 (25.6)
**2-18**	347 (200-660)	33 (5.1)	358 (84-730)	197 (23.1)	0.148	353 (86-678)	230 (15.4)
**19-30**	384 (189-776)	72 (21.7)	539 (228-1008)	202 (51.8)	0.002	446 (200-914)	274 (38)
**31-50**	557 (221-1100)	182 (37.8)	641 (207-1320)	416 (67.5)	0.023	588 (213-1107)	598 (54.5)
**51-70**	689 (219-1650)	172 (57.3)	687 (213-1361)	327 (77.5)	0.094	689 (219-1530)	499 (69.1)
**71+**	551 (180-1141)	25 (75.8)	675 (237-1350)	33 (86.8)	0.600	576 (180-1141)	58 (81.7)
**19+**	560 (200-1105)	451 (39.3)	623 (214-1320)	978 (66.7)	0.003	582 (207-1150)	1429 (54.7)

	Metro and Regional		Remote		P	Total	
**Age Group (yrs)**	**Mean* (g) (10th-90th %ile)**	**N** (%)**	**Mean* (g) (10th-90th %ile)**	**N** (%)**	**Mean*** (Non-remote vs Remote)**	**Mean* (g) (10th-90th %ile)**	**N** (%)**
**Total**	540 (200-1081)	484 (27)	566 (180-1141)	1175 (50.7)	0.268	550 (200-1100)	1659 (40.4)
**Coffee**							
**2-3**	-	-	-	-	-	-	-
**4-8**	-****	-****	-****	-****	-****	-****	-****
**9-13**	-****	-****	-****	-****	-****	-****	-****
**14-18**	388 (239-734)	25 (16.3)	-****	-****	-****	391 (239-734)	33 (9.9)
**2-18**	380 (239-734)	28 (4.3)	-****	-****	-****	368 (203-559)	42 (2.8)
**19-30**	521 (230-1036)	119 (35.8)	380 (220-630)	76 (19.5)	0.126	509 (230-1036)	195 (27)
**31-50**	643 (208-1122)	260 (53.9)	590 (228-1309)	167 (27.1)	0.545	638 (208-1166)	427 (38.9)
**51-70**	522 (200-1057)	177 (59)	378 (80-910)	120 (28.4)	0.014	499 (200-1057)	297 (41.1)
**71+**	-****	-****	-****	-****	-****	376 (181-966)	23 (32.4)
**19+**	572 (208-1101)	572 (49.9)	469 (205-1018)	370 (25.2)	0.027	561 (208-1101)	942 (36.1)
**Total**	560 (216-1077)	600 (33.5)	456 (203-1008)	384 (16.6)	0.005	549 (208-1064)	984 (23.9)
**Fruit Juice/Drink**							
**2-3**	283 (131-520)	42 (41.2)	252 (144-416)	70 (50.7)	0.963	276 (131-520)	112 (46.7)
**4-8**	318 (130-593)	80 (37.4)	261 (130-400)	119 (39.5)	0.915	309 (130-593)	199 (38.6)
**9-13**	351 (158-656)	69 (39.2)	289 (131-520)	82 (35.2)	0.290	341 (131-624)	151 (36.9)
**14-18**	674 (208-2993)	49 (32)	381 (156-624)	49 (27.4)	0.670	634 (208-1638)	98 (29.5)
**2-18**	404 (131-666)	240 (37.2)	289 (131-525)	320 (37.6)	0.314	385 (131-666)	560 (37.4)
**19-30**	641 (158-1234)	79 (23.8)	424 (210-624)	68 (17.4)	0.538	608 (158-1050)	147 (20.4)
**31-50**	443 (210-749)	67 (13.9)	423 (156-728)	72 (11.7)	0.215	439 (208-728)	139 (12.7)
**51-70**	338 (158-450)	31 (10.3)	331 (208-630)	45 (10.7)	0.419	336 (158-473)	76 (10.5)
**71+**	-****	-****	-****	-****	-****	-****	-****
**19+**	552 (158-1050)	178 (15.5)	409 (208-692)	186 (12.7)	0.607	528 (198-1050)	364 (13.9)
**Total**	464 (156-1050)	418 (23.3)	338 (146-624)	506 (21.8)	0.685	443 (156-936)	924 (22.5)

	Metro and Regional		Remote		P	Total	
**Age Group (yrs)**	**Mean* (g) (10th-90th %ile)**	**N** (%)**	**Mean* (g) (10th-90th %ile)**	**N** (%)**	**Mean*** (Non-remote vs Remote)**	**Mean* (g) (10th-90th %ile)**	**N** (%)**
**Cordial**							
**2-3**	357 (158-630)	22 (21.6)	354 (153-783)	32 (23.2)	0.920	356 (158-756)	54 (22.5)
**4-8**	382 (147-788)	50 (23.4)	299 (147-473)	77 (25.6)	0.165	367 (147-735)	127 (24.7)
**9-13**	479 (147-914)	25 (14.2)	462 (147-945)	54 (23.2)	0.762	474 (147-914)	79 (19.3)
**14-18**	914 (357-1377)	22 (14.4)	773 (210-2100)	31 (17.3)	0.656	885 (263-1418)	53 (16)
**2-18**	496 (158-1050)	119 (18.4)	444 (147-945)	194 (22.8)	0.714	485 (158-1050)	313 (20.9)
**19-30**	599 (263-1050)	41 (12.3)	624 (263-1050)	75 (19.2)	0.676	606 (263-1050)	116 (16.1)
**31-50**	732 (210-1575)	51 (10.6)	475 (210-735)	92 (14.9)	0.225	670 (210-1512)	143 (13)
**51-70**	-****	-****	391 (210-525)	30 (7.1)	-****	453 (263-525)	47 (6.5)
**71+**	-****	-****	-****	-****	-****	-****	-****
**19+**	649 (210-1418)	112 (9.8)	531 (222-872)	199 (13.6)	0.219	616 (210-1103)	311 (11.9)
**Total**	558 (210-1103)	231 (12.9)	486 (210-945)	393 (17)	0.544	541 (210-1050)	624 (15.2)
**Soft Drinks**							
**2-3**	247 (78-369)	18 (17.6)	152 (16-390)	23 (16.7)	0.022	220 (52-369)	41 (17.1)
**4-8**	448 (125-780)	60 (28)	411 (146-894)	60 (19.9)	0.844	443 (125-780)	120 (23.3)
**9-13**	529 (260-1040)	66 (37.5)	583 (208-1170)	75 (32.2)	0.450	537 (260-1040)	141 (34.5)
**14-18**	714 (260-1618)	76 (49.7)	750 (312-1300)	85 (47.5)	0.061	719 (260-1498)	161 (48.5)
**2-18**	556 (200-979)	220 (34.1)	555 (146-1300)	243 (28.6)	0.788	556 (200-1000)	463 (30.9)
**19-30**	623 (260-1224)	169 (50.9)	702 (302-1300)	162 (41.5)	0.185	638 (260-1300)	331 (45.8)
**31-50**	629 (260-1123)	183 (38)	812 (312-1404)	196 (31.8)	0.001	666 (260-1200)	379 (34.5)
**51-70**	614 (312-1123)	84 (28)	456 (312-750)	78 (18.5)	0.007	589 (312-1118)	162 (22.4)
**71+**	-****	-****	-****	-****	-****	-****	-****
**19+**	622 (260-1125)	443 (38.6)	719 (312-1300)	441 (30.1)	0.013	640 (260-1200)	884 (33.8)
**Total**	597 (250-1100)	663 (37)	665 (211-1300)	684 (29.5)	0.051	609 (250-1124)	1347 (32.8)
**Plain Milk**							

	Metro and Regional		Remote		P	Total	
**Age Group (yrs)**	**Mean* (g) (10th-90th %ile)**	**N** (%)**	**Mean* (g) (10th-90th %ile)**	**N** (%)**	**Mean*** (Non-remote vs Remote)**	**Mean* (g) (10th-90th %ile)**	**N** (%)**
**2-3**	502 (144-1030)	49 (48)	299 (82-515)	45 (32.6)	0.039	473 (135-1030)	94 (39.2)
**4-8**	259 (93-412)	48 (22.4)	189 (52-268)	59 (19.6)	0.194	249 (82-412)	107 (20.8)
**9-13**	279 (5-412)	31 (17.6)	299 (206-412)	30 (12.9)	0.878	282 (5-412)	61 (14.9)
**14-18**	-****	-****	-****	-****	-****	301 (93-822)	30 (9)
**2-18**	346 (93-822)	144 (22.3)	252 (82-515)	148 (17.4)	0.083	332 (93-773)	292 (19.5)
**19-30**	380 (129-597)	27 (8.1)	190 (30-412)	24 (6.2)	0.008	345 (52-597)	51 (7.1)
**31-50**	359 (32-618)	30 (6.2)	377 (21-1020)	27 (4.4)	0.952	362 (32-680)	57 (5.2)
**51-70**	322 (62-728)	20 (6.7)	-****	-****	-****	313 (62-728)	35 (4.8)
**71+**	-****	-****	-****	-****	-****	-****	-****
**19+**	358 (62-721)	78 (6.8)	273 (30-520)	69 (4.7)	0.130	343 (52-680)	147 (5.6)
**Total**	349 (82-773)	222 (12.4)	259 (32-515)	217 (9.4)	0.028	336 (82-773)	439 (10.7)
**Flavoured Milk**							
**2-3**	-****	-****	-****	-****	-****	286 (60-948)	31 (12.9)
**4-8**	350 (148-748)	51 (23.8)	354 (85-795)	41 (13.6)	0.683	351 (147-748)	92 (17.9)
**9-13**	343 (205-530)	30 (17)	314 (214-515)	21 (9)	0.652	338 (213-530)	51 (12.5)
**14-18**	398 (200-670)	26 (17)	-****	-****	-****	392 (190-670)	40 (12)
**2-18**	353 (131-721)	123 (19.1)	317 (97-636)	91 (10.7)	0.354	349 (131-720)	214 (14.3)
**19-30**	470 (248-556)	38 (11.4)	484 (236-795)	30 (7.7)	0.296	472 (248-556)	68 (9.4)
**31-50**	433 (212-742)	56 (11.6)	638 (268-1272)	25 (4.1)	0.041	450 (212-742)	81 (7.4)
**51-70**	-****	-****	-****	-****	-****	452 (231-636)	23 (3.2)
**71+**	-****	-****	-	-	-****	-****	-****
**19+**	447 (217-636)	106 (9.2)	540 (229-854)	67 (4.6)	0.009	459 (217-670)	173 (6.6)
**Total**	394 (149-670)	229 (12.8)	415 (159-795)	158 (6.8)	0.471	397 (149-680)	387 (9.4)
**Alcohol**							
**2-3**	-	-	-	-	-	-	-

	Metro and Regional		Remote		P	Total	
Age Group (yrs)	Mean* (g) (10th-90th %ile)	N** (%)	Mean* (g) (10th-90th %ile)	N** (%)	Mean*** (Non-remote vs Remote)	Mean* (g) (10th-90th %ile)	N** (%)
4-8	-	-	-	-	-	-	-
9-13	-	-	-	-	-	-	-
14-18	-****	-****	-****	-****	-****	-****	-****
2-18	-****	-****	-****	-****	-****	-****	-****
19-30	1724 (309-4632)	50 (15.1)	2676 (428-7628)	46 (11.8)	0.079	1883 (309-4632)	96 (13.3)
31-50	1678 (248-4545)	115 (23.9)	2906 (379-5681)	102 (16.6)	0.017	1881 (250-4545)	217 (19.8)
51-70	1339 (240-2314)	74 (24.7)	1801 (299-4553)	64 (15.2)	0.092	1409 (277-2828)	138 (19.1)
71+	-****	-****	-****	-****	-****	-****	-****
19+	1593 (250-4524)	245 (21.4)	2554 (379-5681)	218 (14.9)	0.001	1749 (277-4545)	463 (17.7)
Total	1634 (253-4524)	250 (14)	2551 (379-5681)	223 (9.6)	0.001	1783 (287-4545)	473 (11.5)
**Water**							
2-3	749 (150-1500)	76 (74.5)	544 (150-1000)	118 (85.5)	0.036	701 (150-1400)	194 (80.8)
4-8	732 (190-1500)	188 (87.9)	880 (200-1800)	283 (94)	0.118	760 (190-1500)	471 (91.5)
9-13	983 (250-2000)	153 (86.9)	1202 (250-3000)	211 (90.6)	0.007	1029 (250-2000)	364 (89)
14-18	1228 (290-2400)	120 (78.4)	1365 (290-2680)	155 (86.6)	0.733	1255 (290-2400)	275 (82.8)
2-18	935 (200-2000)	537 (83.3)	1059 (250-2000)	767 (90.1)	0.031	960 (200-2000)	1304 (87.2)
19-30	1493 (350-3000)	267 (80.4)	1491 (375-2500)	335 (85.9)	0.491	1492 (350-3000)	602 (83.4)
31-50	1396 (350-3000)	372 (77.2)	1361 (330-2550)	524 (85.1)	0.431	1388 (350-3000)	896 (81.6)
51-70	1108 (200-2250)	224 (74.7)	1218 (330-2100)	352 (83.4)	0.345	1135 (200-2250)	576 (79.8)
71+	788 (240-1740)	29 (87.9)	1268 (450-2000)	34 (89.5)	0.372	874 (240-2000)	63 (88.7)
19+	1366 (330-3000)	892 (77.8)	1375 (350-2500)	1245 (84.9)	0.441	1368 (330-3000)	2137 (81.8)
Total	1177 (250-2320)	1429 (79.7)	1251 (300-2250)	2012 (86.8)	0.015	1194 (250-2320)	3441 (83.8)

*Weighted mean

**Sample N

***P-values using weighted Mann-Whitney U Test at significance level of 0.05

****Summary data is not provided when cell count <20 individuals

Supplementary Table S2: Mean percentage contribution to energy, calcium, vitamin C, and total sugar intake of the population on the day of the National Aboriginal and Torres Strait Islander Nutrition and Physical Activity Survey (NATSINPAS) 2012-13 by remoteness category.

	Percentage of Energy				Percentage of Calcium				Percentage of Vitamin C				Percentage of Total Sugar			
**Age Group (yrs)**	**Metro / Regional**	**Remote**	**P****	**Total**	**Metro / Regional**	**Remote**	**P****	**Total**	**Metro / Regional**	**Remote**	**P****	**Total**	**Metro / Regional**	**Remote**	**P****	**Total**
**All Beverages**																
**2-3**	20.8	14.7	0.031	19.5	37.6	28.3	0.042	35.7	28.6	33.5	0.893	29.6	42.3	41.1	0.689	42.0
**4-8**	12.8	11.1	0.252	12.5	21.7	22.5	0.766	21.8	34.7	32.6	0.987	34.3	37.1	36.6	0.710	37.0
**9-13**	12.1	11.7	0.390	12.0	18.7	21.6	0.052	19.3	29.5	25.5	0.888	28.7	39.3	40.7	0.683	39.6
**14-18**	17.0	15.1	0.924	16.7	25.6	23.2	0.701	25.2	30.1	23.2	0.163	28.8	51.7	50.4	0.977	51.5
**2-18**	14.6	12.8	0.137	14.3	23.7	23.1	0.671	23.6	31.3	28.1	0.306	30.7	42.2	42.1	0.930	42.2
**19-30**	19.3	19.4	0.926	19.3	26.1	29.7	0.193	26.9	31.3	24.9	0.163	30.0	55.4	58.6	0.368	56.0
**31-50**	22.1	19.9	0.023	21.6	32.8	32.2	0.966	32.7	20.4	21.0	0.571	20.6	51.1	51.9	0.919	51.3
**51-70**	16.9	14.9	0.737	16.5	28.1	28.1	0.809	28.1	16.4	18.6	0.066	16.9	34.5	39.5	0.015	35.7
**71+**	11.1	15.9	0.640	12.0	22.9	36.1	0.640	25.2	6.4	9.0	0.591	6.9	26.3	35.8	0.341	28.0
**19+**	19.8	18.6	0.149	19.5	29.1	30.5	0.319	29.4	23.6	21.7	0.669	23.2	49.1	51.3	0.041	49.6
**Total**	17.6	16.4	0.070	17.4	26.9	27.7	0.276	27.0	26.9	24.1	0.584	26.3	46.2	47.8	0.056	46.6
**Tea**																
**2-3**	-***	-***	-***	-***	-***	-***	-***	-***	-***	-***	-***	-***	-***	-***	-***	-***
**4-8**	-***	0.8	-***	0.3	-***	2.7	-***	0.9	-***	0.0	-***	<0.1	-***	2.7	-***	0.9
**9-13**	-***	1.6	-***	0.5	-***	5.2	-***	1.4	-***	0.9	-***	0.2	-***	7.4	-***	2.0
**14-18**	-***	3.2	-***	0.9	-***	8.2	-***	2.1	-***	0.7	-***	0.1	-***	11.6	-***	3.2
**2-18**	0.2	1.7	<0.001	0.5	0.5	4.7	<0.001	1.3	0.0	0.5	<0.001	0.1	0.7	6.3	<0.001	1.7
**19-30**	0.7	4.6	<0.001	1.5	1.6	10.6	<0.001	3.4	0.0	0.5	<0.001	0.1	2.6	14.2	<0.001	4.9
**31-50**	1.7	4.5	<0.001	2.3	4.1	11.3	<0.001	5.7	0.2	2.1	<0.001	0.7	5.8	17.3	<0.001	8.4
**51-70**	2.4	4.9	0.013	3.0	5.7	11.2	0.021	7.0	0.5	2.3	<0.001	0.9	7.1	17.8	0.003	9.6
**71+**	2.1	5.4	1.000	2.7	7.1	16.4	0.242	8.8	1.3	0.7	0.689	1.2	6.9	18.3	0.273	9.0
**19+**	1.5	4.6	<0.001	2.2	3.5	11.1	<0.001	5.2	0.2	1.6	<0.001	0.5	4.8	16.3	<0.001	7.3
**Total**	0.9	3.5	<0.001	1.5	2.2	8.7	<0.001	3.6	0.1	1.2	<0.001	0.3	3.1	12.5	<0.001	5.0

	Percentage of Energy				Percentage of Calcium				Percentage of Vitamin C				Percentage of Total Sugar			
**Age Group (yrs)**	**Metro / Regional**	**Remote**	**P****	**Total**	**Metro / Regional**	**Remote**	**P****	**Total**	**Metro / Regional**	**Remote**	**P****	**Total**	**Metro / Regional**	**Remote**	**P****	**Total**
**Coffee**																
**2-3**	-	-	-	-	-	-	-	-	-	-	-	-	-	-	-	-
**4-8**	-***	-***	-***	-***	-***	-***	-***	-***	-***	-***	-***	-***	-***	-***	-***	-***
**9-13**	-***	-***	-***	-***	-***	-***	-***	-***	-***	-***	-***	-***	-***	-***	-***	-***
**14-18**	0.9	-***	-***	0.7	3.3	-***	-***	2.8	0.0	-***	-***	<0.1	3.8	-***	-***	3.2
**2-18**	0.3	-***	-***	0.2	0.9	-***	-***	0.8	0.0	-***	-***	<0.1	1.1	-***	-***	0.9
**19-30**	2.6	0.8	<0.001	2.2	7.0	2.6	<0.001	6.1	0.6	0.0	0.089	0.5	7.6	2.1	<0.001	6.5
**31-50**	4.9	1.9	<0.001	4.2	12.4	5.2	<0.001	10.8	0.2	0.6	0.936	0.3	12.6	6.0	<0.001	11.1c
**51-70**	3.3	1.5	0.007	2.9	10.9	5.0	0.007	9.5	0.3	0.7	0.014	0.4	9.6	5.8	0.132	8.7
**71+**	-***	-***	-***	2.0	-***	-***	-***	6.4	-***	-***	-***	0.2	-***	-***	-***	4.6
**19+**	3.7	1.4	<0.001	3.2	9.9	4.2	<0.001	8.7	0.4	0.4	0.879	0.4	9.9	4.5	<0.001	8.8
**Total**	2.2	0.9	<0.001	2.0	6.2	2.7	<0.001	5.5	0.2	0.2	0.243	0.2	6.2	2.9	<0.001	5.6
**Fruit Juice/Drink**																
**2-3**	2.2	3.9	0.417	2.6	1.1	2.2	0.169	1.3	21.4	27.1	0.542	22.6	8.7	15.6	0.321	10.1
**4-8**	3.1	2.6	0.789	3.0	1.7	1.6	0.768	1.7	26.8	25.0	0.882	26.5	11.2	11.0	0.936	11.2
**9-13**	3.0	1.8	0.055	2.8	2.5	1.3	0.045	2.3	25.8	20.1	0.100	24.7	11.7	8.0	0.080	10.9
**14-18**	3.2	1.8	0.047	2.9	2.6	1.3	0.040	2.3	22.7	14.4	0.024	21.1	11.9	7.1	0.034	11.0
**2-18**	3.0	2.3	0.039	2.9	2.1	1.5	0.038	2.0	24.8	21.0	0.037	24.1	11.2	9.6	0.060	10.9
**19-30**	2.7	1.3	0.040	2.4	2.2	1.3	0.086	2.0	22.6	15.1	0.048	21.1	10.1	5.8	0.031	9.3
**31-50**	1.1	0.8	0.044	1.1	0.9	0.7	0.035	0.9	9.0	7.0	0.040	8.5	4.0	3.3	0.045	3.9
**51-70**	0.6	0.7	0.509	0.6	0.5	0.8	0.491	0.5	5.8	6.4	0.507	6.0	2.3	3.6	0.522	2.6
**71+**	-***	-***	-***	-***	-***	-***	-***	-***	-***	-***	-***	-***	-***	-***	-***	-***
**19+**	1.6	1.0	0.010	1.5	1.3	0.9	0.012	1.2	13.5	9.6	0.010	12.7	6.0	4.2	0.009	5.6
**Total**	2.2	1.5	<0.001	2.0	1.7	1.1	<0.001	1.5	18.3	13.9	<0.001	17.3	8.2	6.3	<0.001	7.8
**Cordial**																
**2-3**	2.4	1.7	0.353	2.2	0.3	0.3	0.447	0.3	3.3	3.8	0.296	3.4	7.2	6.1	0.350	7.0

	Percentage of Energy				Percentage of Calcium				Percentage of Vitamin C				Percentage of Total Sugar			
**Age Group (yrs)**	**Metro / Regional**	**Remote**	**P****	**Total**	**Metro / Regional**	**Remote**	**P****	**Total**	**Metro / Regional**	**Remote**	**P****	**Total**	**Metro / Regional**	**Remote**	**P****	**Total**
**4-8**	2.0	1.8	0.602	2.0	0.4	0.4	0.692	0.4	4.2	4.6	0.799	4.2	7.6	8.4	0.631	7.8
**9-13**	1.1	2.2	0.051	1.3	0.3	0.6	0.036	0.3	1.9	2.9	0.127	2.1	3.9	8.6	0.045	4.9
**14-18**	1.6	1.9	0.543	1.7	0.5	0.6	0.536	0.5	3.3	4.4	0.448	3.5	5.2	6.4	0.539	5.4
**2-18**	1.7	1.9	0.657	1.7	0.4	0.5	0.540	0.4	3.2	4.0	0.596	3.4	5.9	7.6	0.629	6.2
**19-30**	1.0	1.6	0.157	1.1	0.4	0.5	0.174	0.4	2.3	3.9	0.111	2.6	4.2	6.6	0.141	4.6
**31-50**	1.0	0.9	0.744	1.0	0.3	0.4	0.793	0.3	1.8	2.1	0.417	1.8	3.7	4.5	0.788	3.9
**51-70**	0.4	0.8	0.097	0.5	0.1	0.2	0.091	0.1	1.0	2.3	0.090	1.3	1.7	2.6	0.103	1.9
**71+**	-***	-***	-***	-***	-***	-***	-***	-***	-***	-***	-***	-***	-***	-***	-***	-***
**19+**	0.9	1.1	0.167	0.9	0.3	0.4	0.161	0.3	1.8	2.8	0.021	2.0	3.4	4.8	0.151	3.7
**Total**	1.2	1.4	0.312	1.3	0.3	0.4	0.249	0.3	2.4	3.2	0.089	2.6	4.5	5.9	0.279	4.8
**Soft Drinks**																
**2-3**	0.8	1.0	0.154	0.8	0.1	0.2	0.073	0.1	0.0	0.0	1.000	<0.1	3.2	4.2	0.140	3.4
**4-8**	2.3	1.9	0.506	2.2	0.3	0.4	0.402	0.3	0.0	0.0	1.000	<0.1	8.8	7.6	0.572	8.6
**9-13**	3.7	2.7	0.021	3.5	0.6	0.5	0.037	0.6	0.0	0.0	1.000	<0.1	15.4	9.5	0.023	14.2
**14-18**	5.1	5.3	0.611	5.2	0.8	1.3	0.380	0.9	0.0	0.0	1.000	<0.1	19.4	19.3	0.607	19.4
**2-18**	3.3	2.9	0.128	3.2	0.5	0.6	0.103	0.6	0.0	0.0	1.000	<0.1	12.9	10.9	0.154	12.5
**19-30**	5.3	5.1	0.730	5.2	1.0	1.1	0.961	1.0	0.0	0.0	1.000	<0.1	20.4	20.9	0.623	20.5
**31-50**	3.2	4.1	0.931	3.5	0.8	1.1	0.727	0.8	0.0	0.0	1.000	<0.1	12.6	14.7	0.744	13.1
**51-70**	2.5	1.4	0.382	2.2	0.6	0.4	0.050	0.5	0.0	0.0	1.000	<0.1	8.3	6.2	0.501	7.8
**71+**	-***	-***	-***	-***	-***	-***	-***	-***	-***	-***	-***	-***	-***	-***	-***	-***
**19+**	3.9	3.9	0.862	3.9	0.8	0.9	0.415	0.8	0.0	0.0	1.000	<0.1	14.7	14.9	0.564	14.7
**Total**	3.6	3.5	0.477	3.6	0.7	0.8	0.141	0.7	0.0	0.0	1.000	<0.1	13.9	13.4	0.733	13.8
**Plain Milk**																
**2-3**	11.4	5.9	0.017	10.3	25.8	17.7	0.040	24.2	0.0	0.0	1.000	<0.1	16.3	10.8	0.031	15.1
**4-8**	1.8	1.1	0.050	1.7	6.6	4.6	0.056	6.2	0.0	0.0	0.713	<0.1	2.7	1.9	0.045	2.6
**9-13**	1.5	1.4	0.223	1.5	5.7	5.7	0.255	5.7	0.0	0.1	0.020	<0.1	2.5	2.6	0.275	2.5

	Percentage of Energy				Percentage of Calcium				Percentage of Vitamin C				Percentage of Total Sugar			
**Age Group (yrs)**	**Metro / Regional**	**Remote**	**P****	**Total**	**Metro / Regional**	**Remote**	**P****	**Total**	**Metro / Regional**	**Remote**	**P****	**Total**	**Metro / Regional**	**Remote**	**P****	**Total**
**14-18**	-***	-***	-***	0.8	-***	-***	-***	3.0	-***	-***	-***	<0.1	-***	-***	-***	1.5
**2-18**	2.6	1.6	0.001	2.4	7.6	6.0	0.002	7.3	0.0	0.0	0.142	<0.1	3.8	3.2	0.002	3.7
**19-30**	0.6	0.3	0.205	0.5	2.0	1.4	0.200	1.9	0.0	0.0	0.374	<0.1	0.9	0.7	0.214	0.8
**31-50**	0.5	0.4	0.767	0.5	1.9	1.4	0.760	1.8	0.0	0.0	0.231	<0.1	1.1	0.8	0.752	1.0
**51-70**	-***	-***	-***	0.6	-***	-***	-***	2.3	-***	-***	-***	<0.1	-***	-***	-***	1.4
**71+**	-***	-***	-***	-***	-***	-***	-***	-***	-***	-***	-***	-***	-***	-***	-***	-***
**19+**	0.6	0.4	0.148	0.5	2.1	1.4	0.144	1.9	0.0	0.0	0.130	<0.1	1.1	0.7	0.149	1.0
**Total**	1.4	0.8	<0.001	1.3	4.4	3.1	<0.001	4.1	0.0	0.0	0.403	<0.1	2.3	1.7	<0.001	2.1
**Flavoured Milk**																
**2-3**	-***	-***	-***	3.1	-***	-***	-***	6.6	-***	-***	-***	3.1	-***	-***	-***	5.5
**4-8**	2.8	2.4	0.585	2.7	8.2	6.7	0.637	8.0	3.2	1.8	0.864	2.9	5.2	3.9	0.577	5.0
**9-13**	2.1	1.4	0.366	2.0	5.3	3.4	0.321	4.9	1.3	1.1	0.730	1.3	4.0	2.5	0.336	3.7
**14-18**	2.1	-***	-***	1.9	7.2	-***	-***	6.4	2.3	-***	-***	2.0	4.4	-***	-***	3.8
**2-18**	2.5	1.5	0.017	2.3	7.0	4.3	0.015	6.5	2.5	1.3	0.109	2.2	4.8	2.7	0.014	4.4
**19-30**	1.7	1.3	0.396	1.6	5.0	3.7	0.391	4.7	1.8	0.8	0.345	1.6	2.7	3.0	0.436	2.7
**31-50**	2.0	0.7	0.003	1.7	4.6	2.0	0.004	4.0	2.4	0.4	0.012	2.0	4.2	1.5	0.003	3.6
**51-70**	-***	-***	-***	0.5	-***	-***	-***	1.3	-***	-***	-***	0.3	-***	-***	-***	1.0
**71+**	-***	-	-***	-***	-***	-	-***	-***	-***	-	-***	-***	-***	-	-***	-***
**19+**	1.6	0.9	0.018	1.4	4.0	2.5	0.021	3.7	1.7	0.6	0.026	1.5	2.9	2.0	0.021	2.7
**Total**	2.0	1.1	<0.001	1.8	5.3	3.2	0.001	4.9	2.0	0.8	0.005	1.8	3.7	2.3	0.001	3.4
**Alcohol**																
**2-3**	-	-	-	-	-	-	-	-	-	-	-	-	-	-	-	-
**4-8**	-	-	-	-	-	-	-	-	-	-	-	-	-	-	-	-
**9-13**	-	-	-	-	-	-	-	-	-	-	-	-	-	-	-	-
**14-18**	-***	-***	-***	-***	-***	-***	-***	-***	-***	-***	-***	-***	-***	-***	-***	-***
**2-18**	-***	-***	-***	-***	-***	-***	-***	-***	-***	-***	-***	-***	-***	-***	-***	-***

	Percentage of Energy				Percentage of Calcium				Percentage of Vitamin C				Percentage of Total Sugar			
**Age Group (yrs)**	**Metro / Regional**	**Remote**	**P****	**Total**	**Metro / Regional**	**Remote**	**P****	**Total**	**Metro / Regional**	**Remote**	**P****	**Total**	**Metro / Regional**	**Remote**	**P****	**Total**
**19-30**	3.5	3.8	0.458	3.6	1.1	1.5	0.462	1.2	3.8	3.8	0.778	3.8	3.3	3.5	0.677	3.4
**31-50**	6.9	6.1	0.033	6.7	3.1	4.0	0.051	3.3	6.7	8.8	0.539	7.2	4.8	3.0	0.097	4.4
**51-70**	6.2	4.6	0.110	5.8	2.7	2.1	0.080	2.6	8.6	6.2	0.092	8.1	2.5	1.3	0.036	2.2
**71+**	-***	-***	-***	-***	-***	-***	-***	-***	-***	-***	-***	-***	-***	-***	-***	-***
**19+**	5.4	5.0	0.010	5.3	2.2	2.7	0.012	2.3	5.9	6.4	0.147	6.0	3.7	2.8	0.025	3.5
**Total**	3.4	3.2	0.042	3.3	1.4	1.7	0.048	1.5	3.5	4.3	0.470	3.7	2.4	1.8	0.092	2.3

* All percentages are population weighted

** P-values using weighted Mann-Whitney U Test at significance level of 0.05

***Summary data is not provided when cell count <20 individuals
